# Trends in Disease Severity and Health Care Utilization During the Early Omicron Variant Period Compared with Previous SARS-CoV-2 High Transmission Periods — United States, December 2020–January 2022

**DOI:** 10.15585/mmwr.mm7104e4

**Published:** 2022-01-28

**Authors:** A. Danielle Iuliano, Joan M. Brunkard, Tegan K. Boehmer, Elisha Peterson, Stacey Adjei, Alison M. Binder, Stacy Cobb, Philip Graff, Pauline Hidalgo, Mark J. Panaggio, Jeanette J. Rainey, Preetika Rao, Karl Soetebier, Susan Wacaster, ChinEn Ai, Vikas Gupta, Noelle-Angelique M. Molinari, Matthew D. Ritchey

**Affiliations:** ^1^CDC COVID-19 Emergency Response Team; ^2^Johns Hopkins University Applied Physics Laboratory, Laurel, Maryland; ^3^Booze Allen Hamilton, McLean, Virginia; ^4^Becton, Dickinson and Company, Franklin Lake, New Jersey.

The B.1.1.529 (Omicron) variant of SARS-CoV-2, the virus that causes COVID-19, was first clinically identified in the United States on December 1, 2021, and spread rapidly. By late December, it became the predominant strain, and by January 15, 2022, it represented 99.5% of sequenced specimens in the United States* (*1*). The Omicron variant has been shown to be more transmissible and less virulent than previously circulating variants (*2*,*3*). To better understand the severity of disease and health care utilization associated with the emergence of the Omicron variant in the United States, CDC examined data from three surveillance systems and a large health care database to assess multiple indicators across three high–COVID-19 transmission periods: December 1, 2020–February 28, 2021 (winter 2020–21); July 15–October 31, 2021 (SARS-CoV-2 B.1.617.2 [Delta] predominance); and December 19, 2021–January 15, 2022 (Omicron predominance). The highest daily 7-day moving average to date of cases (798,976 daily cases during January 9–15, 2022), emergency department (ED) visits (48,238), and admissions (21,586) were reported during the Omicron period, however, the highest daily 7-day moving average of deaths (1,854) was lower than during previous periods. During the Omicron period, a maximum of 20.6% of staffed inpatient beds were in use for COVID-19 patients, 3.4 and 7.2 percentage points higher than during the winter 2020–21 and Delta periods, respectively. However, intensive care unit (ICU) bed use did not increase to the same degree: 30.4% of staffed ICU beds were in use for COVID-19 patients during the Omicron period, 0.5 percentage points lower than during the winter 2020–21 period and 1.2 percentage points higher than during the Delta period. The ratio of peak ED visits to cases (event-to-case ratios) (87 per 1,000 cases), hospital admissions (27 per 1,000 cases), and deaths (nine per 1,000 cases [lagged by 3 weeks]) during the Omicron period were lower than those observed during the winter 2020–21 (92, 68, and 16 respectively) and Delta (167, 78, and 13, respectively) periods. Further, among hospitalized COVID-19 patients from 199 U.S. hospitals, the mean length of stay and percentages who were admitted to an ICU, received invasive mechanical ventilation (IMV), and died while in the hospital were lower during the Omicron period than during previous periods. COVID-19 disease severity appears to be lower during the Omicron period than during previous periods of high transmission, likely related to higher vaccination coverage,^†^ which reduces disease severity (*4*), lower virulence of the Omicron variant (*3*,*5*,*6*), and infection-acquired immunity (*3*,*7*). Although disease severity appears lower with the Omicron variant, the high volume of ED visits and hospitalizations can strain local health care systems in the United States, and the average daily number of deaths remains substantial.^§^ This underscores the importance of national emergency preparedness, specifically, hospital surge capacity and the ability to adequately staff local health care systems. In addition, being up to date on vaccination and following other recommended prevention strategies are critical to preventing infections, severe illness, or death from COVID-19.

CDC used data from three surveillance systems to assess U.S. disease related to COVID-19 during December 1, 2020–January 15, 2022. COVID-19 aggregate cases and deaths reported to CDC by state and territorial health departments^¶^ were tabulated by report date.** ED visits with COVID-19 diagnosis codes were obtained from the National Syndromic Surveillance Program (NSSP).^††^ Hospital admissions and inpatient and ICU bed use among patients with laboratory-confirmed COVID-19 were obtained from the Unified Hospital Data Surveillance System.^§§^ ED visits and hospital admissions were tabulated by admission date and stratified by the following age groups: 0–17, 18–49, and ≥50 years.

The maximum 7-day moving averages of the daily number of COVID-19 cases, ED visits, hospital admissions, and deaths during the Omicron period were compared with the peak 7-day moving averages for the winter 2020–21 and Delta periods. The maximum percentages of inpatient and ICU bed use overall and by COVID-19 patients were compared between periods. For each period analyzed, ratios of ED visits, hospital admissions, and deaths per 1,000 COVID-19 cases were calculated.^¶¶^

CDC used the BD Insights Research Database (BD), a U.S. health care facility database,*** to assess hospitalized COVID-19 patients as a percentage of total hospital admissions: the percentage of hospitalized COVID-19 patients who were admitted to an ICU, received IMV, or died while in the hospital; and the mean and median length of hospital stay.^†††^ Indicators were tabulated based on discharge date and stratified by age group: 0–17, 18–50, and >50 years.^§§§^ Three-week windows were analyzed during each period to stabilize estimates.^¶¶¶^ Statistical differences between the Omicron and winter 2020–21 and Delta periods were assessed using z-tests for proportions and t-tests for mean length of stay; statistical significance criterion was p<0.05.

Analyses were carried out in Python (version 3.8.6, Python Software Foundation) and Kotlin (version 1.4, Kotlin Foundation).**** This activity was reviewed by CDC and conducted consistent with applicable federal law and CDC policy.^††††^

The daily 7-day moving average of COVID-19 cases, ED visits, and hospital admissions rapidly increased during the Omicron period (Figure). However, during the week ending January 15, 2022, ED visits appeared to be decreasing and the rapid increase in cases and hospital admissions appeared to be slowing. As of January 15, 2022, the maximum daily 7-day moving average number of cases (798,976), ED visits (48,238), admissions (21,586), and deaths (1,854) observed during the Omicron period reflects changes of 219%, 137%, 31%, and −46%, respectively, compared with those during the winter 2020–21 period, and 386%, 86%, 76%, and –4%, respectively, compared with those during the Delta period (Table 1). The largest relative differences in ED visits and admissions were observed among children and adolescents aged 0–17 years during the Omicron period; however, this age group represented only 14.5% of COVID-19 ED visits and 4.2% of COVID-19 admissions. During the Omicron period, a maximum of 20.6% of staffed inpatient beds were in use for COVID-19 patients, 3.4 and 7.2 percentage points higher than during the winter 2020–21 and Delta periods, respectively. However, ICU bed use did not increase to the same degree: 30.4% of staffed ICU beds were in use for COVID-19 patients during the Omicron period, 0.5 percentage points lower than during the winter 2020–21 period and 1.2 percentage points higher than during the Delta period. When comparing the indicators at their peaks during the Omicron period, event-to-case ratios for ED visits (87 visits per 1,000 cases), hospitalizations (27 hospitalizations per 1,000 cases), and deaths (nine deaths per 1,000 cases [lagged by 3 weeks]) were lower than those observed during the peak winter 2020–21 (92, 68, and 16, respectively) and Delta (167, 78, and 13, respectively) periods (Supplementary Figure, https://stacks.cdc.gov/view/cdc/113628).

**FIGURE:**
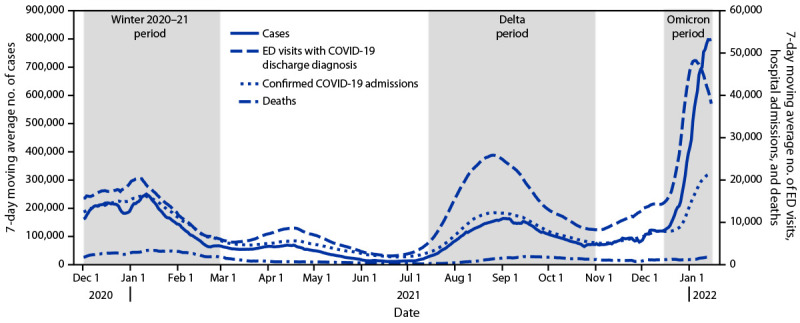
Seven-day moving average number of COVID-19 cases, emergency department visits, hospital admissions, and deaths — United States,* December 1, 2020–January 15, 2022 **Sources:** CDC state-reported data (cases and deaths), Unified Hospital dataset (admissions), and National Syndromic Surveillance Program (ED visits with COVID-19 discharge diagnoses). **Abbreviation:** ED = emergency department. * COVID-19 hospital admissions include admissions for COVID-19 as well as patients who receive a positive SARS-CoV-2 test result after being admitted for other reasons. National Syndromic Surveillance Program represents approximately 70% of all U.S. ED visits.

**TABLE 1:** COVID-19 disease, hospital, and death indicators during the Omicron period compared with the winter 2020–21 and Delta periods* — United States, December 2020–January 2022^†^

Indicator/Age group, yrs	Winter 2020–21 period		Delta period		Omicron period		Comparison of Omicron with winter 2020–21 period		Comparison of Omicron with Delta period	
	Peak value date range	Peak value (7-day moving average)	Peak value date range	Peak value (7-day moving average)	Date of maximum assessed value^§^	Maximum 7-day moving average	Number or percentage point difference^¶^	Relative % difference**	Number or percentage point difference^¶^	Relative % difference**
**Disease (cases, ED visits)**										
**COVID-19 cases, N**	Jan 4–11, 2021	250,335	Aug 25–Sep 1, 2021	164,249	Jan 15, 2022	798,976	548,641	219.2	634,727	386.4
**COVID-19 ED visits, by age group, N (% of total)**	Dec 29, 2020–Jan 5, 2021	20,372	Aug 19–26, 2021	25,873	Jan 4, 2022	48,238	27,866	136.8	22,365	86.4
0–17		901 (4.4)		3,177 (12.3)		6,990 (14.5)	6,089 (10.1)	676.1	3,813 (2.2)	120.0
18–49		6,872 (33.7)		11,853 (45.8)		23,372 (48.5)	16,500 (14.7)	240.1	11,519 (2.6)	97.2
≥50		12,406 (60.9)		10,546 (40.8)		17,471 (36.2)	5,066 (−24.7)	40.8	6,926 (−4.5)	65.7
**Hospital (admissions)**										
**COVID-19 admissions, by age group, N (% of total)**	Jan 2–9, 2021	16,497	Aug 20–27, 2021	12,285	Jan 15, 2022	21,586	5,089	30.8	9,301	75.7
0–17		207 (1.3)		319 (2.6)		914 (4.2)	707 (3.0)	341.9	595 (1.6)	186.5
18–49		2,761 (16.7)		3,559 (29.0)		5,218 (24.2)	2,457 (7.4)	89.0	1,659 (−4.8)	46.6
≥50		12,840 (77.8)		7,828 (63.7)		14,773 (68.4)	1,933 (−9.4)	15.1	6,945 (4.7)	88.7
**Inpatient beds in use for COVID-19, N**	Jan 4–11, 2021	125,100	Aug 28–Sep 4, 2021	94,503	Jan 15, 2022	142,687	17,587	14.1	48,184	51.0
**Staffed beds in use for COVID-19, %**		17.2		13.4		20.6	3.4	20.0	7.2	53.7
**Staffed beds in use, %**		74.1		76.8		79.2	5.1	6.9	2.4	3.1
**ICU beds in use for COVID-19, N**	Jan 9–16, 2021	27,958	Sep 6–13, 2021	24,774	Jan 15, 2022	24,776	−3,182	−11.4	2	0.0
**Staffed ICU beds in use for COVID-19, %**		30.9		29.2		30.4	−0.5	−1.7	1.2	4.2
**Staffed ICU beds in use, %**		78.2		79.6		82.2	4.0	5.1	2.6	3.2
**Deaths**										
**COVID-19 deaths, N**	Jan 6–13, 2021	3,422	Sep 9–15, 2021	1,924	Jan 15, 2022	1,854	−1,568	−45.8	−70	−3.6

**Sources:** CDC state-reported data (case and death totals), CDC case line-level data (cases by age), Unified Hospital data set (hospital admissions, inpatient, and ICU), and National Syndromic Surveillance Program (ED visits with COVID-19 discharge diagnoses).

**Abbreviations:** ED = emergency department; ICU = intensive care unit; N = no. of hospital admissions.

* COVID-19 hospital admissions include admissions for COVID-19 as well as patients who receive a positive test result for COVID-19 after being admitted for other reasons. National Syndromic Surveillance Program data are not inclusive of all ED visits, representing approximately 71% of all visits. The peak value and associated date are calculated independently for each indicator as the highest 7-day moving average value during Dec 1, 2020–Jan 31, 2021 (winter 2020–21 period), Aug 1–Sep 30, 2021 (Delta period), or Dec 19, 2021–Jan 15, 2022 (Omicron period). The date and value of peaks might change slightly if data are backfilled.

^†^ Data were pulled on January 20, 2022.

^§^ Maximum value date for the Omicron period was assessed for December 19, 2021–January 15, 2022. This date is defined as the maximum value for each of the severity indicators at the time that the data were pulled for this report on January 20, 2022. The date of the maximum value might be different at the time of publication.

^¶^ Total difference is presented for the number of cases, ED visits, hospital admissions, deaths, and inpatient and ICU beds in use. Percentage point difference is presented for the percentage of ED visits and hospital admissions by age groups and for the percentages of inpatient and ICU beds in use for COVID-19 patients.

** Relative percent difference is calculated as the value for cases, ED visits, hospital admissions, inpatient bed use, ICU bed use, and deaths from the Omicron period minus the same indicator value from the comparison period (winter 2020–21 or Delta period) divided by the same indicator value from the comparison period.

In BD, hospitalized COVID-19 patients represented 12.0%, 9.4%, and 12.9% of all admissions during the winter 2020–21, Delta, and Omicron periods, respectively. Disease severity among hospitalized COVID-19 patients was associated with increasing age; IMV and in-hospital deaths were rare among patients aged 0–17 years, therefore, differences between periods were not assessed. The percentage of hospitalized COVID-19 patients admitted to an ICU during Omicron (13.0%) was 28.8% lower than during the winter 2020–21 (18.2%) and 25.9% lower than during Delta (17.5%) periods overall, and for all three age groups (p<0.05) (Table 2). The percentage of hospitalized COVID-19 patients who received IMV (3.5%) or died while in the hospital (7.1%) during Omicron was lower than during the winter 2020–21 (IMV = 7.5%; deaths = 12.9%) and Delta (IMV = 6.6%; deaths = 12.3%) periods overall, and for both adult age groups (p<0.001). Mean length of hospital stay during Omicron (5.5 days) was 31.0% lower than during the winter 2020–21 (8.0 days) and 26.8% lower than during Delta (7.6 days) periods overall, and for both adult age groups (p<0.001).

**TABLE 2:** Total hospitalizations, hospitalized COVID-19 patients, and indicators of disease severity among hospitalized COVID-19 patients during the Omicron period compared with the winter 2020–21 and Delta periods,* by age group, 199 hospitals—United States, January 2021–January 2022

Indicator/Age group, yrs	No. (%)			Comparison of Omicron with winter 2020–21 period		Comparison of Omicron with Delta period	
	Winter 2020–21 period	Delta period	Omicron period				
	Jan 1–21, 2021	Aug 22–Sep 11, 2021	Dec 26, 2021–Jan 15, 2022	Percentage point or mean difference	Relative % difference	Percentage point or mean difference	Relative % difference
**Total hospitalizations**							
All	108,360	110,950	98,920	—	—	—	—
0–17	11,504	13,946	11,517	—	—	—	—
18–50	31,070	34,537	28,040	—	—	—	—
>50	65,786	62,467	59,363	—	—	—	—
**Hospitalized COVID-19 patients as a percentage of total hospitalizations**							
All	12,963 (12.0)	10,440 (9.4)	12,800 (12.9)	1.0^†^	8.2	3.5^†^	37.5
0–17	147 (1.3)	272 (2.0)	405 (3.5)	2.2^†^	175.2	1.6^†^	80.3
18–50	2,474 (8.0)	3,304 (9.6)	3,988 (14.2)	6.3^†^	78.6	4.7^†^	48.7
>50	10,342 (15.7)	6,864 (11.0)	8,407 (14.2)	−1.6^†^	−9.9	3.2^†^	28.9
**ICU admission among hospitalized COVID-19 patients**							
All	2,359 (18.2)	1,824 (17.5)	1,658 (13.0)	−5.2^†^	−28.8	−4.5^†^	−25.9
0–17	25 (17.0)	50 (18.4)	42 (10.4)	−6.6**^§^**	−39.0	−8.0**^§^**	−43.6
18–50	346 (14.0)	438 (13.3)	377 (9.5)	−4.5^†^	−32.4	−3.8^†^	−28.7
>50	1,988 (19.2)	1,336 (19.5)	1,239 (14.7)	−4.5^†^	−23.3	−4.7^†^	−24.3
**IMV among hospitalized COVID-19 patients** ^¶^							
All	764 (7.5)	503 (6.6)	358 (3.5)	−4.0^†^	−53.4	−3.1^†^	−46.5
0–17	1 (0.8)	1 (0.4)	0 (—)	NC	NC	NC	NC
18–50	122 (6.2)	118 (4.9)	73 (2.3)	−3.9^†^	−63.2	−2.6^†^	−53.2
>50	641 (8.0)	384 (7.7)	285 (4.3)	−3.7^†^	−46.2	−3.4^†^	−44.3
In-hospital death among hospitalized COVID-19 patients**							
All	976 (12.9)	803 (12.3)	533 (7.1)	−5.8^†^	−44.9	−5.2^†^	−42.3
0–17	1 (1.1)	0 (—)	0 (—)	NC	NC	NC	NC
18–50	57 (4.0)	110 (5.4)	38 (1.7)	−2.3^†^	−58.3	−3.7^†^	−69.2
>50	918 (15.2)	693 (16.0)	495 (10.0)	−5.2^†^	−34.2	−6.0^†^	−37.5
**Length of stay among hospitalized COVID-19 patients, by age group, yrs**							
**Median**							
All	5	5	3	—	—	—	—
0–17	2	2	2	—	—	—	—
18–50	3	4	2	—	—	—	—
>50	5	6	4	—	—	—	—
**Mean (SD)**							
All	8.0 (15.6)	7.6 (10.6)	5.5 (13.1)	−2.5^†^	−31.0	−2.0^†^	−26.8
0–17	4.4 (10.1)	3.9 (5.3)	3.5 (9.7)	−0.9	−20.3	−0.4	−9.5
18–50	5.8 (7.8)	6.1 (6.9)	4.3 (7.4)	−1.5^†^	−25.6	−1.8^†^	−29.9
>50	8.6 (17.0)	8.4 (12.0)	6.2 (15.1)	−2.4^†^	−27.7	−2.2^†^	−25.8

**Source:** BD Insights Research Database.

**Abbreviations:** ICU = intensive care unit; IMV = invasive mechanical ventilation; NC = not calculated.

*The winter period was defined as January 1–21, 2021, the Delta period was defined as August 22–September 11, 2021, and the Omicron period was defined as December 26, 2021–January 15, 2022 for BD analysis.

^†^ p<0.001.

**^§^** p<0.05.

^¶^ Data on IMV were available from a subset of 135 hospitals. The denominators of hospitalized COVID-19 patients for IMV percentages were as follows for each period and age group: winter 2020–21 (0–17 years [132]; 18–50 years [1,964]; and >50 years [8,039]); Delta (0–17 years [258]; 18–50 years [2,415]; and >50 years [4,988]); and Omicron (0–17 years [355]; 18–50 years [3,189]; and >50 years [6,646]).

** Data on in-hospital deaths were available from a subset of 148 hospitals. The denominators of hospitalized COVID-19 patients for in-hospital death percentages were as follows for each period and age group: winter 2020–21 (0–17 years [87]; 18–50 years [1,437]; and >50 years [6,048]); Delta (0–17 years [142]; 18–50 years [2,045]; and >50 years [4,333]); and Omicron (0–17 years [250]; 18–50 years [2,297]; and >50 years [4,954]).

## Discussion

Emergence of the Omicron variant in December 2021 led to a substantial increase in COVID-19 cases in the United States. Although the rapid rise in cases has resulted in the highest number of COVID-19–associated ED visits and hospital admissions since the beginning of the pandemic, straining the health care system, disease severity appears to be lower than compared with previous high disease-transmission periods. In addition to lower ratios of ED visits, hospitalizations, and deaths to cases observed during the Omicron period, disease severity indicators were also lower among hospitalized COVID-19 patients, including ICU admission, receipt of IMV, length of stay, and in-hospital death. This apparent decrease in disease severity is likely related to multiple factors, most notably increases in vaccination coverage among eligible persons (*4*,*8*), and the use of vaccine boosters among recommended subgroups^§§§§^ (*9*). For example, during the Omicron period, 207 million persons were fully vaccinated compared with 178 million persons and 1.5 million persons during the Delta and the winter 2020–21 periods, respectively (*8*). Further, during the Omicron period, 78 million persons had received vaccine boosters compared with 1.6 million persons during the Delta period; boosters were not available during winter 2020–21 (*8*). Other key factors for lower disease severity include infection-acquired immunity (*3*,*7*), and potential lower virulence of the Omicron variant (*3*,*5*,*6*).

These findings are consistent with reports from South Africa (*2*), England (*10*), and Scotland,^¶¶¶¶^ as well as from health systems in California (*3*) and Texas,***** where the Omicron variant was not associated with an increase in hospital or disease severity indicators among patients with Omicron infections compared with those with Delta infections. Death and in-hospital severity indicators, including in the context of vaccination status, should continue to be monitored for changes or differential effects among subpopulations throughout the Omicron period.

Among children aged <18 years, in-hospital severity indicators, including length of stay and ICU admission, were similar to and lower, respectively, during the Omicron period compared with those during previous high-transmission periods. However, high relative increases in ED visits and hospitalizations were observed among children during the Omicron period, which might be related to lower vaccination rates in children compared with those in adults, especially among children aged 0–4 years who are currently not eligible for vaccination. Children’s susceptibility to the Omicron variant and the impact of changes in exposure on severity risk require additional study. Among adults aged ≥18 years, all in-hospital severity indicators assessed were lower during the Omicron period, which might be related to increased population immunity against SARS-CoV-2 because of higher vaccination coverage and booster rates and previous infection providing protection (*3*,*4*,*7*,*9*). Receipt of a third mRNA vaccine dose was found to be highly effective at preventing urgent care encounters, ED visits, and hospital admissions during both Delta and Omicron periods (*9*). Booster doses were also found to be effective at preventing infection during the early Omicron period, particularly among persons aged ≥50 years (*4*).

The findings in this report are subject to at least seven limitations. First, BD is not nationally representative and NSSP does not capture all ED visits across the United States; therefore, geographic and demographic differences in disease transmission and severity might bias findings. Second, the variation in vaccination coverage during the three periods assessed was not taken into account when comparing severity indicators. This limitation is most relevant when comparing the Omicron period to the winter 2020-21 period, when vaccines were just becoming available in the United States. Third, person-level vaccination status was not available to compare severity indicators based on being up to date on vaccinations. Fourth, the hospital data do not exclude incidental SARS-CoV-2 infections, which might be higher during the Omicron period because of increased transmissibility of the Omicron variant; inclusion of incidental infections could inflate hospitalization-to-case ratios and have an unknown effect on in-hospital severity indicators. Fifth, changes in testing and reporting behaviors, including the likely increase in self-administered tests, might bias comparisons; specifically, reported case counts during the Omicron period might be biased downward because of self-administered test use compared with counts during other periods.^†††††^ Sixth, co-circulation of the Omicron and Delta variants might affect the magnitude of the severity indicators during the beginning of the Omicron period, particularly for in-hospital severity indicators based on date of hospital discharge. Finally, the findings reflect an ecologic analysis of event-based indicators; findings should not be misinterpreted as person-level indicators (e.g., case-fatality ratios).

Emergence of the Omicron variant has resulted in a rapid increase in COVID-19 cases. Concurrent increases in ED visits and hospital admissions appear to be driven by high case counts and not by increased disease severity following acute infection. Although patients hospitalized during the Omicron period have shorter stays and less frequent ICU admissions, the high volume of hospitalizations resulting from high transmission rates during a short period can strain local health care systems in the United States, and the average daily number of deaths remains substantial. This underscores the importance of national emergency preparedness, specifically, hospital surge capacity and the ability to adequately staff local health care systems when critical care needs arise and before the system is overwhelmed. Previous studies have identified increased risk for severe outcomes among unvaccinated persons (*4*,*9*). Thus, being up to date with COVID-19 vaccinations and following other recommended prevention strategies are critical to prevent infections, severe illness, or death from COVID-19.

**Summary**

**What is already known about this topic?**

The SARS-CoV-2 B.1.1.529 (Omicron) variant became predominant in the United States by late December 2021, leading to a surge in COVID-19 cases and associated ED visits and hospitalizations.

**What is added by this report?**

Despite Omicron seeing the highest reported numbers of COVID-19 cases and hospitalizations during the pandemic, disease severity indicators, including length of stay, ICU admission, and death, were lower than during previous pandemic peaks.

**What are the implications for public health practice?**

Although disease severity appears lower with the Omicron variant, the high volume of hospitalizations can strain local health care systems and the average daily number of deaths remains substantial. This underscores the importance of national emergency preparedness, specifically, hospital surge capacity and the ability to adequately staff local health care systems. In addition, being up to date on vaccinations and following other recommended prevention strategies are critical to preventing infections, severe illness, or death from COVID-19.

## Footnotes

* Predominance defined as >50% of specimens sequenced. Proportion that was Omicron variant during the week ending December 18: 39.4%; December 25: 71.6%; January 1: 92.3%; January 8: 98.3%; and January 15: 99.5%. https://covid.cdc.gov/covid-data-tracker/#variant-proportions

† https://covid.cdc.gov/covid-data-tracker/#vaccinations_vacc-total-admin-rate-total (Accessed January 15, 2022).

§ https://covid.cdc.gov/covid-data-tracker/#trends_dailydeaths (Accessed January 15, 2022).

¶ CDC official counts of COVID-19 cases and deaths, released daily (https://covid.cdc.gov/covid-data-tracker), are aggregate counts from reporting jurisdictions. A COVID-19 case is defined by detection of SARS-CoV-2 RNA or antigen in a respiratory specimen collected from a person with a confirmed or probable case of COVID-19 according to the Council of State and Territorial Epidemiologists’ updated case definition. https://cdn.ymaws.com/www.cste.org/resource/resmgr/ps/ps2021/21-ID-01_COVID-19.pdf

** Date of report is used for consistency because most jurisdictions are not reporting case by onset or test date. The same applies to deaths, where there might be an even larger lag between date of death and date of report of death.

†† NSSP collects electronic health data, including ED visits with COVID-19 diagnoses, from a subset of hospitals in 49 states and the District of Columbia (71% of nonfederal EDs in the United States). ED visits for COVID-19 are defined as ED visits with any of the following: *International Classification of Diseases, Tenth Revision* codes U07.1 or J12.82 or Systematized Nomenclature of Medicine codes 840539006, 840544004, or 840533007. https://www.cdc.gov/nssp/overview.html

§§ The U.S. Department of Health and Human Services (HHS) Unified Hospital Data Surveillance System includes data from all U.S. hospitals registered with the Centers for Medicare & Medicaid Services (CMS) as of June 1, 2020, and from hospitals not CMS–registered but reporting COVID-19 data through this system since July 1, 2020. Data, including counts of new hospital admissions of patients with confirmed COVID-19 by age group, are reported to HHS either directly from facilities or via a state health department submission; on January 11, 2022, 96% of hospitals reported data. This analysis includes children’s, short-term acute care, long-term acute care, critical access, Veterans Administration, Defense Health Agency, and Indian Health Services hospitals and excludes psychiatric, rehabilitation, and religious nonmedical hospitals. Reporting guidelines are published in the HHS COVID-19 Guidance for Hospital Reporting and FAQs document. https://www.hhs.gov/sites/default/files/covid-19-faqs-hospitals-hospital-laboratory-acute-care-facility-data-reporting.pdf

¶¶ Ratios of ED visits and hospital admissions per 1,000 COVID-19 cases were calculated as peak daily 7-day moving averages of ED visits or hospital admissions divided by the corresponding daily 7-day moving average of COVID-19 cases observed when peak ED visits and hospital admissions occurred. The ratio of deaths per 1,000 COVID-19 cases was calculated with a 3-week lag in cases from the date when peak daily 7-day moving averages of deaths occurred to account for the time between case ascertainment, occurrence of death, and reporting.

*** BD is a large U.S. health care facility database that includes patient-level electronically captured laboratory results; pharmacy orders; and admission, discharge, and transfer data from 267 hospitals. CDC receives aggregate BD data via a surveillance dashboard that is updated every Tuesday with data for the previous week (Sunday through Saturday) (release date January 18, 2022; access date January 18, 2022). This analysis used data from 199 hospitals that reported data during all three periods; of these hospitals, 135 (68%) had information for IMV, and 148 (74%) had information on in-hospital deaths. Geographically, hospital distribution is uneven; hospitals are overrepresented in the South (53%) and Northeast (17%) U.S. Census regions and underrepresented in the West (9%) and Midwest (21%) regions. Monthly trends in disease severity indicators from BD and two other data sources are available on CDC COVID Data Tracker. https://covid.cdc.gov/covid-data-tracker/#hospitalizations-severity

††† Hospitalized COVID-19 patients were identified by the presence of a positive SARS-CoV-2 polymerase chain reaction or antigen test result during the 14 days before or 14 days after date of admission; 43% of all admissions did not have a SARS-CoV-2 test result available (January 2021–January 2022). To identify patients admitted to an ICU, care settings were classified using the CDC National Healthcare Safety Network classification and then further classified as ICU (critical care) or non-ICU (inpatient adult wards, specialty care areas, and step-down wards); https://academic.oup.com/ofid/article/5/10/ofy241/5104818. Because of lack of timely device data to identify ventilator use, the following surrogate definition for IMV use was used: a) the patient was started on intravenous/intravenous push (IV/IVP) sedation medications (propofol, lorazepam, midazolam, dexmedetomidine, or ketamine) or IV/IVP opioids (fentanyl, remifentanil, sufentanil, or hydromorphone) with a duration ≥24 hours, and b) at least two arterial blood gas results were collected at least 24 hours apart (on the first day of sedation medication and a subsequent result 24 hours later) (https://academic.oup.com/ofid/article/8/6/ofab232/6285220). In-hospital death was defined by a designation of death, mortality, or presence in morgue in the admission, discharge, and transfer data feeds. A validity check of this method had been previously performed by randomly selecting 50 mortality cases and evaluating encounter-level data for clinical signs of mortality (e.g., uncorrected severe metabolic acidosis determined by pH from arterial blood gases and uncorrected electrolyte changes incompatible with life). https://jamanetwork.com/journals/jamanetworkopen/fullarticle/2778237

§§§ Age groups for patients are preestablished on the BD surveillance dashboard and do not align exactly with older age grouping (i.e., aged <50 years versus ≥50 years) available in the surveillance data.

¶¶¶ The 7-day daily average peak in the case surveillance data was used as the central day of the 3-week window for the winter 2020–21 (January 1–January 21, 2021) and Delta (August 22–September 11, 2021) periods; the most recent 3 weeks of available data were used for the Omicron period (December 26, 2021–January 15, 2022). Date ranges are based on date of hospital discharge.

**** Surveillance data cleaning, processing, and calculation of peak rates were conducted using Python and Kotlin.

†††† 45 C.F.R. part 46; 21 C.F.R. part 56; 42 U.S.C. Sect. 241(d); 5 U.S.C. Sect. 552a; 44 U.S.C. Sect. 3501 et seq.

§§§§
https://www.cdc.gov/coronavirus/2019-ncov/vaccines/booster-shot.html

¶¶¶¶
https://www.pure.ed.ac.uk/ws/portalfiles/portal/245818096/Severity_of_Omicron_variant_of_concern_and_vaccin%20e_effectiveness_against_symptomatic_disease.pdf%2012

*****
https://www.medrxiv.org/content/10.1101/2021.12.30.21268560v2

††††† Case data in this report are based on data reported by states. Some states report both confirmed and probable cases, and some states report only confirmed cases. For states that include probable cases, a case based on antigen test results with symptoms might meet the case definition and be included in the probable case count. However, positive self-administered tests alone (which are also antigen tests) might not be reported to public health authorities, and do not meet the current Council of State and Territorial Epidemiologists’ case definition criteria, and thus, will likely not be included by states in probable case counts reported to CDC.
